# The Application of an Omentum Graft or Flap in Spinal Cord Injury

**DOI:** 10.3390/ijms22157930

**Published:** 2021-07-25

**Authors:** Li-Yu Fay, Yan-Ru Lin, Dann-Ying Liou, Chuan-Wen Chiu, Mei-Yin Yeh, Wen-Cheng Huang, Jau-Ching Wu, May-Jywan Tsai, Henrich Cheng

**Affiliations:** 1 Institute of Pharmacology, College of Medicine, National Yang Ming Chiao Tung University, No. 155, Sec. 2, Linong St., Taipei 11217, Taiwan; leofay1978@gmail.com (L.-Y.F.); cindyya2002@gmail.com (M.-Y.Y.); wchuang518@gmail.com (W.-C.H.); jauching@gmail.com (J.-C.W.); 2 School of Medicine, National Yang Ming Chiao Tung University, No. 155, Sec. 2, Linong St., Taipei 11217, Taiwan; 3 Department of Neurosurgery, Neurological Institute, Taipei Veterans General Hospital, Room 525, 17F, No. 201, Sec. 2, Shipai Road, Taipei 11217, Taiwan; yanlumiku@gmail.com (Y.-R.L.); dragonspruce@gmail.com (D.-Y.L.); gmgdbaby@gmail.com (C.-W.C.)

**Keywords:** spinal cord injury, omentum, laminin, vascularization, transplantation

## Abstract

Background: Spinal cord injury (SCI) causes a primary injury at the lesion site and triggers a secondary injury and prolonged inflammation. There has been no definitive treatment till now. Promoting angiogenesis is one of the most important strategies for functional recovery after SCI. The omentum, abundant in blood and lymph vessels, possesses the potent ability of tissue regeneration. Methods: The present work examines the efficacy of autologous omentum, either as a flap (with vascular connection intact) or graft (severed vascular connection), on spinal nerve regeneration. After contusive SCI in rats, a thin sheath of omentum was grafted to the injured spinal cord. Results: Omental graft improved behavior scores significantly from the 3rd to 6th week after injury (6th week, 5.5 ± 0.5 vs. 8.6 ± 1.3, *p* < 0.05). Furthermore, the reduction in cavity and the preservation of class III β-tubulin-positive nerve fibers in the injury area was noted. Next, the free omental flap was transposed to a completely transected SCI in rats through a pre-implanted tunnel. The flap remained vascularized and survived well several weeks after the operation. At 16 weeks post-treatment, SCI rats with omentum flap treatment displayed the preservation of significantly more nerve fibers (*p* < 0.05) and a reduced injured cavity, though locomotor scores were similar. Conclusions: Taken together, the findings of this study indicate that treatment with an omental graft or transposition of an omental flap on an injured spinal cord has a positive effect on nerve protection and tissue preservation in SCI rats. The current data highlight the importance of omentum in clinical applications.

## 1. Introduction

Traumatic spinal cord injury (SCI) is one of the most devastating diseases and results in severe motor and sensory dysfunction below the level of injury. It causes primary injury to both neuronal axons and myelin sheaths, which is followed by secondary injury and reactive gliosis, which leads to further neurological damage [1,2,3]. The secondary injury after SCI involves the release of cytotoxic factors, tissue edema, decreased blood flow, and accelerated apoptosis [4,5]. The prolonged inflammatory reaction and limited regenerative ability both restrict the restoration of function. The current strategies for restoration can be divided into neuroprotection and neuroregeneration according to the different time-points, although there is no definitive discrimination. Neuroprotection aims to reduce the inflammatory response, which could induce neurons or supportive cells to undergo apoptosis or necrosis. Neuroregeneration aims to reduce the inhibitory effect of extracellular materials and to promote the regrowth of neurons or axons. Because of the limited regenerative capability and the presence of inhibitory materials at the injury site, effective treatments for SCI are not currently available.

In 1996, Cheng et al. led a study and concluded that adult rats with SCI could have partial recovery of hindlimb function after nerve transfer and acidic fibroblast growth factor (aFGF) [6]. His study group in Taiwan then performed several studies to support the promising therapy [7,8,9,10,11,12,13]. This therapy may be one of the multi-modality treatments for SCI.

The omentum flap is a tissue with many capillaries, which can promote the absorption of tissue edema and blood clotting. The post-injury fibrinogens, which form fibrin scars, are also reduced [14]. Professor Rafael et al. published several studies about the treatment of Cervical Spondylotic Myeloradiculopathy (CSM) with surgical decompression and omental transplantation [15]. Both neurological improvement and a vertebral osseous density increase were noted. Duffill et al. conducted a clinical study of 17 patients with chronic SCI who underwent omental transposition. The interval from injury to transposition ranged between 1.5 and 36 years. They placed the pedicle omentum flap directly on the pia surface of the cord. The results showed a minor improvement without a significant difference [14,16,17,18]. The authors believed that the omental flap may still be applied for increased blood supply. According to the mechanism of omentum transposition, early transposition would be more effective to reduce scar formation.

The current study aimed to find vasogenic evidence of an omentum graft and determine the possible effects of omentum flap transposition in the acute stages of SCI in adult rats. We designed a new technique for omentum transposition and evaluated tissue staining and the Basso–Beattie–Bresnahan (BBB) locomotor score for proof.

## 2. Results

### 2.1. Identification of Rat Omentum

**Figure 1:**
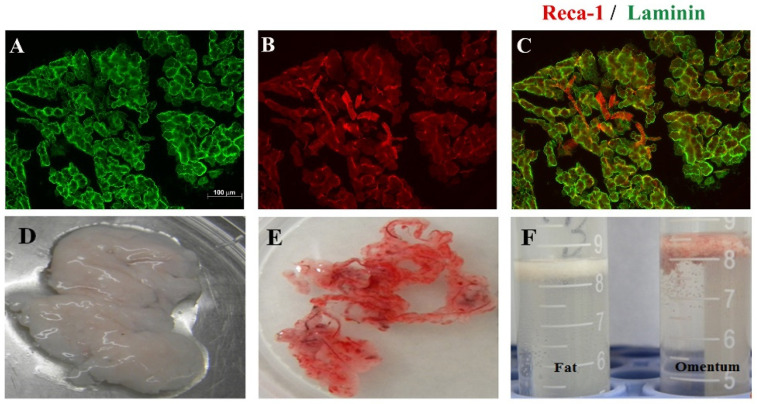
Identification of the rat omentum. (**A**) Laminin-immunoreactive omentum in situ; Scale bar = 100 μm; ×200; (**B**) RECA-1-immuoreactive omentum in situ; (**C**) double-labeling RECA-1 (red)/Laminin (green)-immunoreactive omentum (superimposed photos from **A**,**B**); (**D**) Trimmed fat tissue in dish; (**E**) Trimmed omental tissue in dish; (**F**) Properties of trimmed fat or omentum were suspended in PBS after centrifugation.

Omental tissue is a highly vascularized organ and a rich source of angiogenic factors. A new omental dissection method was designed. The greater omentum consists of a thin double layer of mesothelial cells that is attached to the greater gastric curvature. It folds back on itself and returns to the transverse colon and reaches beyond to the posterior peritoneum. Rat omental pedicle was excised and characterized by immunohistochemical staining. It showed that omental tissue is rich in laminin (green-labeled tissues, Figure 1A) because it was strongly immunoreactive to laminin. Laminin is a glycoprotein which is a constituent of basement membranes [19]. It is also found to promote neuron axonal regeneration after injury [20]. Omental tissue is known to be rich in vascular endothelium. In the present study, the RECA-1-immunoreactive cells (red color, Figure 1B) could be detected. Under the microscope, the rat omentum was marked obviously with laminin and RECA-1. We also found that the omentum is rich in adipocytes, which have a lower density and can be suspended in PBS after centrifugation. This characteristic is quite similar to that of fat tissue (Figure 1).

### 2.2. Beneficial Effect of the Omentum Graft in Contusive SCI Rats

**Figure 2:**
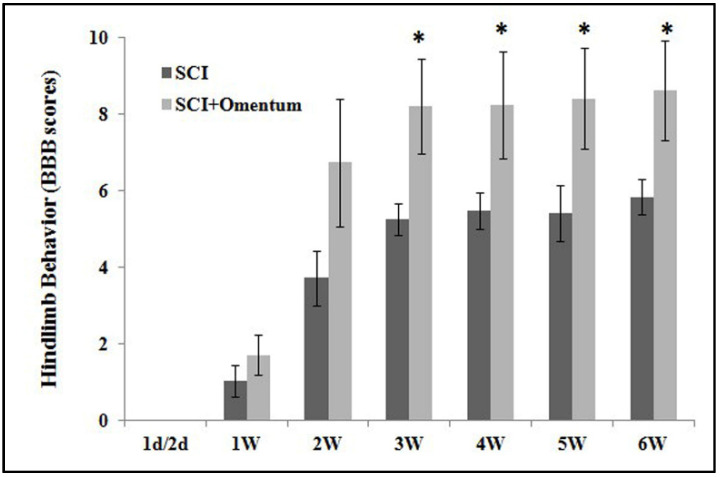
Time course of hindlimb locomotor scores in contusive SCI rats treated with saline or omentum graft. Locomotor recovery of contusive SCI rats was evaluated over a 6-week period, using a 21-point BBB score. The hindlimb recovery of the SCI rats was assessed in a double-blind manner. *n* = 9, 7 rats for groups of SCI, SCI + Omentum, respectively. Results are demonstrated as mean value ± SEM. * *p* < 0.05, SCI versus SCI + Omentum by a one-way ANOVA followed by Bonferroni post hoc test. There were significant differences between the two groups from 3rd to 6th weeks after treatment (*p* < 0.05). The omentum graft demonstrated a beneficial effect in contusive SCI rats.

Traumatic injury to spinal cord initiates a series of cellular and molecular events. Early intervention may limit the extent of tissue injury. Because no resorption of the omentum occurs, it is necessary to trim all tissue and use only the amount necessary to reconstruct the wound. The vascular anatomy of the omentum permits the isolation of a flap within the major omental graft. A piece of omentum graft was implanted to the injury epicenter immediately after eliciting severe contusive SCI in the rats. The hindlimb performance of these SCI rats was monitored weekly up to 6 weeks post-injury. The time course of hindlimb locomotor recovery (the BBB score) in SCI rats treated with saline or omental graft was recorded (Figure 2). Beginning 3 weeks post-treatment onward, the omentum-grafted SCI rats maintained significantly higher BBB scores, indicating functional recovery throughout 6 weeks post-injury (from the 3rd to the 6th week, 5.0 vs. 8.2, 5.2 vs. 8.3, 5.3 vs. 8.4, 5.5 vs. 8.6, *p* < 0.05, respectively).

**Figure 3:**
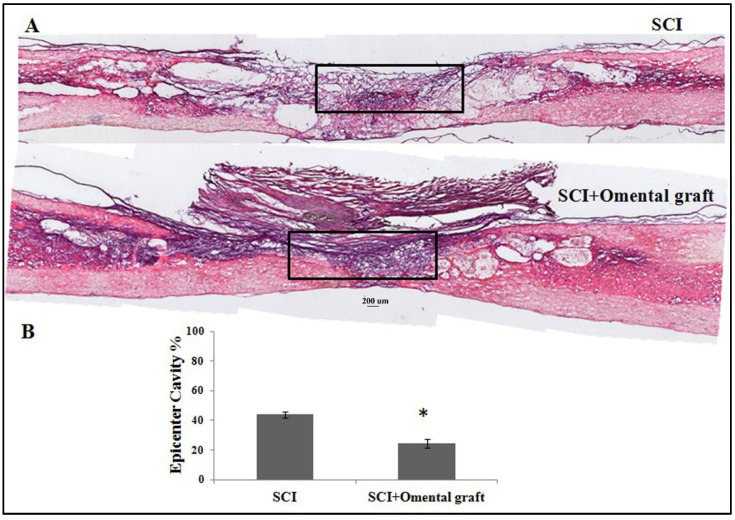
Hematoxylin and eosin (H&E) staining of injured thoracic spinal cords. (**A**) Representative images of longitudinal spinal cord sections in contusive SCI rats with saline or omentum graft at 6 weeks post-injury. (**B**) Quantification of cavities in the injured epicenter (calculated from a rectangle area of 1500 × 300 dpi in the injured cords (shown in **A**)) of contusive SCI rats with saline or omentum graft. All pixels within the rectangles were analyzed with Image J software. Data are expressed as mean value ± SEM. The cavity in contusive SCI rats with omentum graft was significantly smaller (* *p* < 0.05).

**Figure 4:**
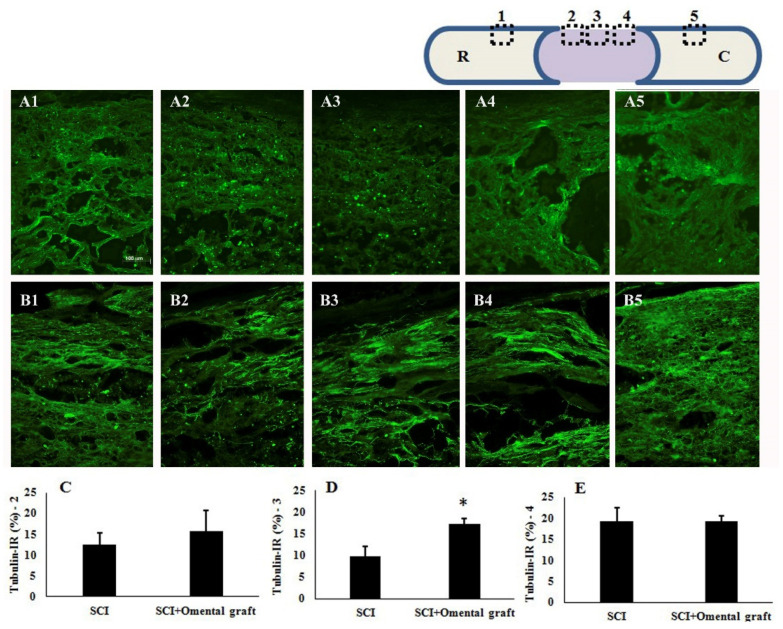
Effect of an omental graft on the morphology of spinal cords in contusive SCI rats at 6 weeks post-injury. (**A1**–**A5**) Figure shows representative class III β-tubulin immunoreactivity (IR) in the injured epicenter of the thoracic spinal cord in contusive SCI rats. Scale bar = 100 μm; ×200; (**B1**–**B5**) Class III β-tubulin IR in the injured spinal cords of omentum-grafted rats. (**C**–**E**) Quantification of class III β-tubulin-immunoreactive axons in areas **A2** (or **B2**), **A3** (or **B3**), and **A4** (or **B4**), respectively, of spinal cord sections. The upper right scheme demonstrates the location of photos **A1**–**A5** and **B1**–**B5** taken from the longitudinal spinal cord section (R: rostral; C: caudal). Note the significant preservation of class III β-tubulin-positive nerve fibers in area B3 of the omentum-treated groups at 6 weeks post-injury (* *p* < 0.05). The results are demonstrated as mean value ± SEM.

At 6 weeks post-injury, the two groups of contusive SCI rats with or without omentum graft were anesthetized and perfused intravascularly with 4% paraformaldehyde. The thoracic regions of the spinal cords were sectioned in the sagittal plane (10 µm thick) and processed for hematoxylin and eosin (H&E) staining as well as IHC. The result showed that the omental graft better preserved the injured spinal cord, compared to SCI without treatment (Figure 3). The quantitative results of H&E staining showed that the omental graft significantly reduced the cavity (43.9 ± 2.1 vs. 24.4 ± 3.0, *p* < 0.05) in the injured epicenter of the spinal cord (Figure 3B). Further histological assessment with immunofluorescent class III β-tubulin staining in longitudinal spinal cord sections was consistent with the results of H&E staining. As shown in Figure 4, the omentum-grafted contused rats had higher axonal densities in the lesions at 6 weeks after SCI (Figure 4B3 compared to Figure 4A3). Quantitative stereological analysis of tissue areas (areas 2, 3, 4) revealed a significant difference in area 3 (9.9 ± 2.3 vs. 17.2 ± 1.4, *p* < 0.05) among the samples studied (Figure 4C–E). The omentum-grafted rat spinal cords possessed more preserved class III β-tubulin (+) nerve fibers than those of contusive SCI rats without treatment.

### 2.3. Vascularity of the Omental Flap and Application in Transected SCI Rats

The omental graft would release progressively fewer angiogenic factors and present poor survival of its vascular network after a prolonged period following transplantation. The use of an omental flap, a well-vascularized organ delivering stem cells, and angiogenic and anti-inflammatory factors for spinal nerve regeneration, was applied. We hypothesized that the omental flap might provide effective coverage and restoration of the contour. An omental flap was transected starting at the hepatic flexure of the colon. Special attention was paid to check the angioarchitecure to ensure that ample blood supply could enter the base of the flap and the tip could be extended from the pedicle. The tubing path and the omentum flap were prepared before making a spinal cord transection injury in the rat. The spinal cord was completely transected at the T-8 level with a 5 mm segment removal. This led to a gap at the transection site that was subsequently filled with adhesive fibrin glue, followed by omental transposition onto the underlying glue bridge.

**Figure 5:**
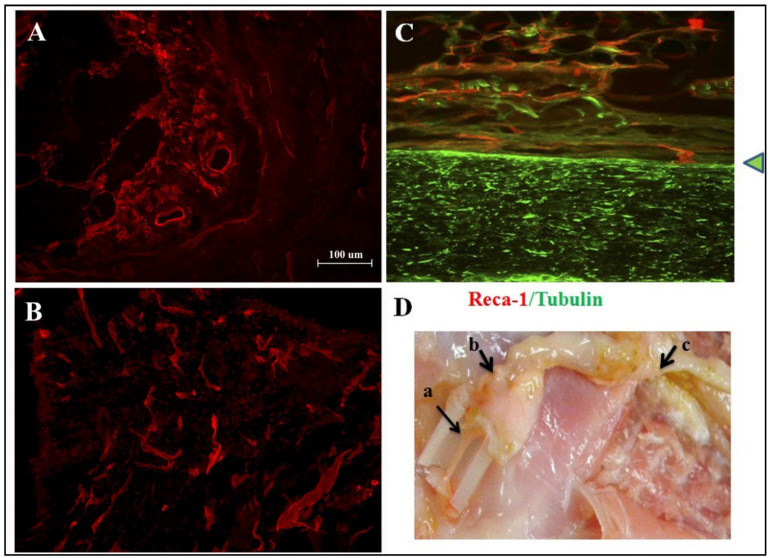
The omental flap remains vascularized at four weeks after transposition to the rat spinal cord. (**A**) RECA-1-immunoreactive omental flap inside the tubing (arrow a in **D**). Scale bar = 100 μm; ×200. (**B**) RECA-1-IR omental flap extruding from the tubing (arrow b in **D**). (**C**) Omentum flap wrapping on the thoracic spinal cord (arrow c in **D**); note that the arrowhead indicates dura matter; red: RECA-1(+) vessel in the omentum; green: tubulin (+) axons. (**D**) The route of omental transposition; the IHC results of a, b, c tissue sections are shown in **A**, **B**, **C**, respectively.

The omentum was temporarily sutured in place and covered with muscle. The rats received careful postoperative care until 16 weeks post-surgery. Two omental flap-treated rats were first examined at 4 weeks post-surgery to check the status of the omental flap in vivo. Three sites in the omental transpositional routes, including the tubing inside and outlet, and the spinal cord wrapping, were all immunoreactive to RECA-1, indicating a vascularized omental flap (Figure 5). The omental flap might remain vascularized after prolonged transposition in vivo. The transected SCI rats with or without omental flap treatment were recorded with BBB score weekly.

**Figure 6:**
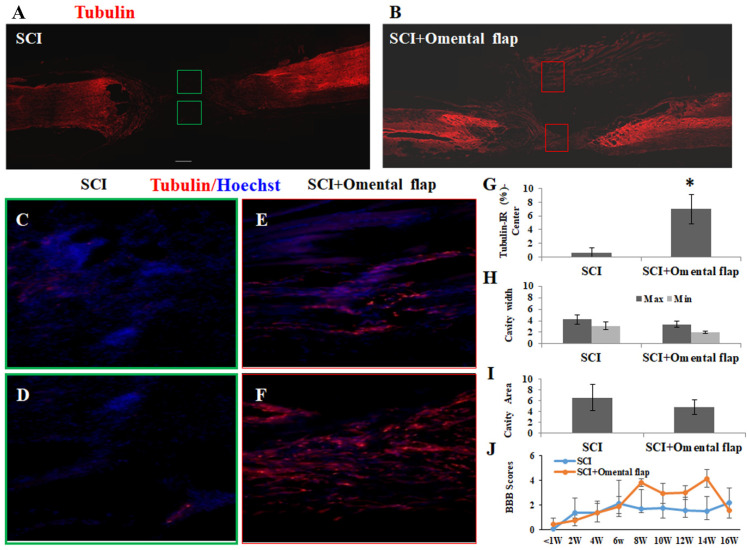
Effect of omental flap on the transected SCI rats. (**A**) Spinal cord section of class III β-tubulin immunoreactivity from transected SCI rats with saline. (**B**) Spinal cord section of class III β-tubulin IR from transected SCI rats with omental flap (magnification_100×) at 16 weeks post-treatment. Left: rostral stump; right: caudal stump of the spinal cord. (**C**,**D**) Class III β-tubulin IR (magnification_200×) from the injured epicenter indicated in the green blocked area of the spinal cord in A. (**E**,**F**) Class III β-tubulin IR (magnification_200×) from the injured epicenter indicated in the red blocked area of the spinal cord in B. (**G**) The quantitative results of class III β-tubulin IR in the injured epicenter of transected SCI rats with saline or omental flap treatment. Omentum flap could preserve more class III β-tubulin in transected SCI rats significantly (*p* < 0.05). (**H**) The width of lesion cavity in the transected SCI rats with saline or omentum flap. There was no significant difference between the two groups. (**I**) There was no significant difference in cavity area in the transected SCI rats with saline or omentum flap. Cavity width and area was calculated after GFAP staining of the longitudinal spinal cord sections. (**J**) The hindlimb functional behaviors, expressed as BBB scores, in SCI rats with saline or omental flap treatment, 4 rats per groups. The results are reported as mean ± SEM. Statistical significance was evaluated using one-way ANOVA and Bonferroni’s t-test. * *p* < 0.05.

At 16 weeks post-treatment, these SCI rats were sacrificed for morphological evaluation. The immunohistochemical results in the spinal cord sections of the transected SCI rats with or without omental flap treatment were compared (Figure 6). The spinal cord sections from both experimental groups were double-stained with anti-beta III tubulin (a neuronal marker) and anti-GFAP (an astroglial marker). The SCI rats with an omental flap demonstrated significantly greater promotion of nerve fiber regeneration (0.67 ± 0.65 vs. 7.0 ± 2.1, *p* < 0.05). The GFAP void cavity width and area in the injured epicenter was reduced in the omental flap-treated SCI rats, although the values did not reach a significant difference. In consideration of the BBB score between the two groups, there was a slight improvement in the omentum flap treatment group, without any significant difference (Figure 6J).

## 3. Discussion

Traumatic SCI causes primary injury to both neuronal axons and myelin sheaths, which is followed by secondary injury with further ongoing neurological damage. The secondary injury after SCI involves the release of cytotoxic factors and molecular reactions. Because of the limited regenerative capability and the presence of inhibitory materials at the injury site, effective treatments for SCI are not currently available. Omentum, a highly vascular organ that is rich in angiogenic factors, may provide a beneficial molecular and cellular environment for regeneration. In the present study, we identified and characterized omental tissues in situ by immunohistochemical study. We further demonstrated the significant regenerative efficacy of autologous omentum, either as an attached (with vascular connection intact, omental flap) or detached tissue (severed vascular connection, omental graft), on spinal nerve regeneration in SCI rats in vivo.

To study the regenerative and angiogenic effects of omental treatments for SCI in rats, we devoted much effort to setting up an omental dissection method and characterization. We found that the omentum is a RECA-1-rich and laminin-rich tissue, as shown in Figure 1. These results were consistent with the reported properties of vascularized omental tissue. Laminin also has been proven to be beneficial for axonal regeneration. Nieuwenhuis et al. confirmed that laminin, the major component of basal lamina, promotes the adhesion, migration, and regeneration of axons [20]. Thus, the existence of a laminin-rich graft or flap would promote the repair of a contusion injury of the spinal cord. This suggests that a flap or graft derived from a well-vascularized omentum may provide a beneficial molecular and cellular environment for regeneration and be suitable for use in a vascularization study.

The omentum is a large mesenchymal fibro-fatty tissue with remarkable healing capability. The stromal cells in omentum are a rich source of growth factors and include omental adipose cells, fibroblasts, pericytes, and leukocytes [21]. Omentum functions to control inflammation and promote revascularization [22,23]. We first optimized and examined the effect of an omental graft in contusive SCI rats, which is highly relevant to injury in the human spinal cord. The dura remained intact after contusive SCI in rats. After eliciting contusion on the thoracic spinal cord, the dura matter was excised in the neighboring cords, allowing the approach of exogenous factors or stem cells to the cords. A piece of omental graft was transferred and wrapped around the injured epicenter. The results in Figure 3, Figure 4 and Figure 5 show that the omental graft improved the behavior scores significantly from the 3rd to 6th week after injury (6th week, 5.5 ± 0.5 vs. 8.6 ± 1.3, *p* < 0.05). The reduction in injury cavity and the preservation of class III β-tubulin-positive nerve fibers in the injury area was noted. These are consistent with the results of Goldsmith et al., who employed intact omentum transposed through a subcutaneous tunnel connected to the laminectomy site of a traumatized feline spinal cord [24]. SCI caused cellular destruction at the lesion site and triggered secondary injury with prolonged inflammation, leading to the irreversible loss of neurological functions. The results of the present study and those of Goldsmith et al. both indicate that omentum might curtail edema or inflammation and promote tissue healing, as suggested in a review article [22].

Because the omental graft would release progressively fewer angiogenic factors and present poor survival of its vascular network after a prolonged grafting period, we next examined the application of an omental flap (with vascular connection intact) for spinal nerve regeneration. An omental flap was transected starting at the hepatic flexure of the colon. The method was similar to the study published by Chooramini et al. in 2013 [25]. At 16 weeks after transection injury with or without omentum transposition, a gap of 5 mm with few tubulin-positive nerve fibers was observed in the transected SCI rat’s spinal cord. By contrast, omental flap treatment had beneficial effects on spinal cord regeneration. The spinal cord tissue was kept highly intact in the omental flap-treated group. The quantitative results of class III β-tubulin immunoreactivity showed a significant difference between the two groups. There were also regenerative nerve fibers across the transection gap. The results conclusively demonstrated that omentum treatment is beneficial for spinal cord regeneration.

The omentum has also been used to support the regeneration of neurons across a freshly transected spinal cord in experiments in cats and also in one patient [26,27]. The spinal cord transection was reconstructed using an omental–collagen bridge in which the transection site was filled with semi-liquid collagen, followed by omental transposition onto the underlying collagen bridge. By incorporating a neuroactive agent such as 4-aminopyridine or laminin into the collagen bridge and combining this with omental transposition, nerve regeneration was evident [26]. The present study simply employed omental transposition to a fibrin-stabled transected spinal cord and obtained beneficial effects. Omentum is a well-vascularized organ delivering different stem cell types and releasing various angiogenic and anti-inflammatory factors. Expression of stem cell markers and angiogenic growth factors has been identified in omentum and this explains some of its regenerative and revascularization properties observed in the present study.

An omentum flap is rich in vascularity and angiogenesis factors, which could decrease the inflammation reaction around the injury site and promote angiogenesis. Goldsmith et al. led several studies that concluded that omentum transposition could decrease lymphedema and improve neurological functioning after traumatic SCI [24,28,29]. Some vasoactive cytokines were also noted in the omentum, which also improved patients’ neurological functions [27,30]. Neurotrophins were also noted in the omentum tissue. It is believed that the neurotrophins not only accumulated in but also were produced by the omentum [31].

In conclusion, the findings of this study indicate that treatment with an omental graft or transposition of an omental flap on an injured spinal cord have a positive effect on nerve protection and tissue preservation in SCI rats. Repair of spinal cord injury is believed to be a multidisciplinary cooperation. Application of different treatments at different time-points may be required to restore neurological function. Based on the current study, optimization of the omentum transposition procedure should be considered. The current data highlight the importance of omentum in clinical applications.

## 4. Materials and Methods

### 4.1. Reagents and Antibodies

Primary antibodies and suppliers were rabbit or mouse anti-neuronal class III β-tubulin (Covance, Cranford, NJ, USA) and rabbit anti-glial fibrillary acidic protein (GFAP) (Dako Cytomation, Ely, Cambridgeshire, UK). Unless stated otherwise, all other chemicals were purchased from Sigma-Aldrich Co (St. Louis, MO, USA).

### 4.2. Omentum Graft or Flap

The omentum, which is known to have rich vasculature, was expected to be a source of blood supply. We identified and prepared an omentum graft or flap for in vivo use in SCI rats. The graft did not have an intact blood supply and therefore relied on the growth of new blood vessels. On the other hand, the flap had an intact blood supply that could supply essential materials immediately. We applied an omental graft or flap to wrap around the injured spinal cord as a support for neurovascular regeneration. An omental flap can be transected starting at the hepatic flexure of the colon. Special attention was paid to ensure that an ample blood supply was entering the base of the flap. Moreover, an appropriate length was needed for the flap to reach the spinal cord without tension. The omental flap was rerouted through pre-implanted tubing, reaching the dorsal side of the spinal cord. The soft tube could work as a tunnel for passage of the flap without incarceration of the omentum pedicle. To ensure the tunnel’s stability, the tube edge was attached to the soft tissue by three Nylon sutures. The soft tube was then connected to the abdominal space and the spinal column. It is important to note that the omental flap for wrapping the injured spinal cord was harvested from the injured animal’s own tissue. Omentum identification and characterization were conducted before being transferred to the injury site. Tissue immunohistochemistry (IHC) staining was performed in the omentum graft group at 4 weeks and in the omentum flap group at 16 weeks after injury in vivo.

### 4.3. Spinal Cord Injury and Treatment

We used Sprague-Dawley (SD) rats, 250–300 g weight, and all surgeries were performed under inhalation anesthesia with isoflurane. The use of animals conformed to the Guiding Principles in the Care and Use of Animals of the American Physiology Society and was approved by the Institutional Animal Care and Use Committee of Taipei Veterans General Hospital. All efforts were made to minimize the number of animals used and their suffering.

We employed two models of SCI rats: a contusive SCI model and a completely transected SCI model. The model of rat contusion injury is highly relevant to injury in the human spinal cord. The dura remains intact after SCI. The present study first optimized and examined the effect of an omental graft in contusive SCI rats. We employed an omental flap transposition in a completely transected SCI rat model.

After being anesthetized, the rats received SCI of contusion utilizing a force-calibrated NYU weight-drop device, following the methods described in our previous reports [32,33]. Briefly, the animals were placed in a stereotaxic frame. The spine was immobilized with a vertebral clamp. A skin incision was made, extending from the mid to low thoracic regions. Laminectomy of the caudal portion of T8 and all of T10 exposed the spinal cord. To inflict a wound on the dorsal surface of the spinal cord, a 10 g weighted rod was dropped from a height of 50 mm onto the exposed dura of the thoracic spinal cord. After spinal cord contusive injury, the rats were assigned to two treatment groups: (1) contusive SCI + saline and (2) contusive SCI + omental graft, in which the injured cord was wrapped with the omentum graft. After treatment, the muscle and skin were closed with interrupted sutures. The contusive SCI rats were kept for 6 weeks.

For conducting the omental transposition experiment in completely transected SCI rats, a tubing path was pre-implanted in the back of the body, near the thoracic spinal cord, allowing the omental flap pedicle to traverse. Subsequently, a 5 mm segment of the thoracic spinal cord was completely removed at level 8 (T8). Fibrin glue was applied to the gap. The eight transected SCI rats were divided into two groups: (1) transected SCI + saline, (2) transected SCI + omentum flap. The omental flap was rerouted through the tubing path to wrap over the T7-10 spinal cord. The muscle and skin were closed with interrupted sutures after treatment.

All the SCI rats recovered in a warmed cage with water and food easily accessible. The rats received careful postoperative care. Bladders were expressed twice a day until spontaneous voiding began. Behavior tests using the BBB locomotor score were conducted weekly after SCI and treatment. At 16 weeks post-injury, SCI rats were transcardially perfused with phosphate-buffered saline (PBS), followed by 4% paraformaldehyde for morphological and biochemical assays. Thoracic spinal cords were then dissected and processed for IHC staining.

### 4.4. Behavioral Examination

The hindlimb locomotor behavior of the rats was evaluated using the BBB locomotor score [34]. Rats were adapted to an open field first. After walking continuously in the open field, the walking pattern of each rat was recorded for 5 min as a digital video. The locomotor function was evaluated by two persons blinded to the rats’ treatment status. The open field locomotor activity score was determined by observation and scoring of behaviors involving the trunk, tail, and hindlimbs. BBB scores ranged from 0 to 21 (0, no movement; 21, normal movement). Hindlimb function was assessed weekly for 8 weeks.

### 4.5. Immunohistochemistry Analysis

At the specified post-injury times, the rats received an overdose of pentobarbital and were perfused intravascularly with 0.9% saline and 4% paraformaldehyde in PBS. The spinal cords were collected, processed, and set into Tissue-Tek O.C.T. Compound (Sakura Finetek, Torrance, CA, USA) on the end of the mounting block. The sample was plunged directly into liquid nitrogen for 10–15 s immediately after securing it to the block. The frozen sample was then removed and stored in a freezer at −80 °C. Sections were cut to a thickness of 10 µm in a cryostat and air-dried. Spinal cord sections were processed for immunostaining with primary antibodies against class III β-tubulin (for all axons), GFAP (for astroglia), anti-endothelial antibody (RECA-1) (for vessels), or laminin. The tissue sections were further incubated with respective secondary antibodies for histological evaluation as previously described [33,35].

### 4.6. Statistical Analysis

Experimental data were expressed as the mean of independent value ± SEM and were analyzed using a one-way analysis of variance (ANOVA) followed by Bonferroni post hoc test. *p* values less than 0.05 were considered statistically significant.

## Data Availability

The data presented in this study are available on request from the corresponding authors.
